# An Analytical Essay on the Scammonies of Aleppo and Smyrna, with Some Observations on the Reddening of Litmus by Resins

**Published:** 1811-02

**Authors:** 


### *An analytical Essay on the Scammonies of Aleppo and Smyrna, with some Observations on the reddening of Litmus by Resins: by Messrs.* Bouillon-Lagrange *and* Vogel.

[Nicholson's Journal.]

The two sorts of scammony are obtained from the root of a plant, that grows in Syria. The finest and purest scammony is procured by making an incision in the root, and drying in the sun the juice that exudes. But frequently, in order to obtain a larger quantity, the people of Syria and Natolia express the juice, and not only from the root, but from the stalks and leaves also. Often too they adulterate it, by mixing with it the juice of some other milky and acrid plants, as that of the spurges; or increase its weight by a mixture of ashes and other foreign matters. To know that the the scammony contains none of these heterogeneous substances, the buyer should break the lumps, choose those that are shining interiorly, and reject those that appear too black, burnt, or containing sand.

Aleppo scammony is light, of an ashen grey, shining, transparent in its fracture. That of Smyrna is very compact, heavy, of a darker colour, and more difficult to powder.

### *Examination of Aleppo scammony.*

When this scammony is pure, it melts entirely on a heated plate of iron, and emits fumes of a nauseating smell. Triturated with water it renders it milky. With boiling water it concretes into a lump: the water becomes yellow, and has a bitter taste; but it is neither alkaline, nor acid; which proves, that this substance is not adulterated with ashes, as some authors affirm.

Alcohol at 40° [sp. grav. 0·817] produces a slight precipitate in this aqueous solution; and the acetat of lead occasions a yellow flocculent precipitate soluble in nitric acid.

The alcoholic tincture of scammony has a brownish yellow colour. It reddens tincture of litmus: and leaves on evaporation a yellowish white and transparent resin.

This resin dissolves entirely in nitric acid, which it colours yellow. The addition of water renders this solution slightly turbid.

It is equally soluble in a solution of pure potash, even without heat, when its colour is yellow: but, if heat be employed, it is brown. Water, even in pretty large quantity, does not precipitate any of the resin. If the solution be saturated with muriatic acid, the resin does not separate. This triple compound of resin, acid, and potash, claims the notice of practitioners; perhaps we may thus discover a solvent for resins, that water would not render turbid.

The part of the scammony that was not soluble in alcohol assumed a grey colour when dry. Treated with boiling water it coloured it yellow, and alcohol occasioned a white flocculent precipitate in it.

To determine the proportion of the constituent principles of Aleppo scammony, we took 100 parts of this substance, and exhausted them by alcohol. The solution was yellow. A grey substance remained, which, when dried, weighed 26.

The alcoholic solution was evaporated to a sirupy consistence. Cold water precipitated from it a resin, which formed a homogeneous mass. The supernatant liquor was clear and colourless. Evaporated to dryness a brown matter was obtained, soluble both in water and in alcohol, and precipitable by by acetate of lead. This substance appeared to be what is called extract. When dried it weighed 2 parts.

The resinous mass, separated and dried, was yellow, and weighed 60.

The 26 parts insoluble in alcohol were then treated with boiling water. After evaporation a glutinous matter remained, weighing 3 parts, and having all the characters of gum. The remainder consisted of fibres of vegetables and a little silex.

The distillation of Aleppo scammony exhibited nothing remarkable. Its products were a very acid brown liquor, and a light, blackish oil. The coal was black, shining, and compact. It contained the carbonates of potash and lime, alumine, silex, and a little iron.

*Examination of Smyrna scammony.*

The fusion of Smyrna scammony is less complete than that of Aleppo. Instead of concreting into a lump with boiling water, it becomes clotty; but the water poured off has similar qualities.

An equal portion of this scammony, exhausted by boiling alcohol, afforded a tincture of a deeper colour, though containing less resin. By evaporation a brownish, transparent resin was obtained, weighing 28 parts. The matter insoluble in alcohol weighed 66. This residuum, treated with boiling water, coloured it yellow. The solution had a faint sweetish taste; and alcohol produced in it a flocculent precipitate soluble in water. On evaporation it left a thick glutinous matter resembling mucilage, soluble with heat in weak nitric acid, and letting fall on cooling a white pulverulent substance, that had all the characters of mucous acid.

In this experiment water took up only 8 parts of the matter insoluble in alcohol. The remainder was subjected to the action of nitric acid assisted by heat, which dissolved it with effervescence. Ammonia added to this solution threw down a precipitate solution in potash. Potash and oxalate of ammonia too occasioned a precipitate. This residuum therefore, beside vegetable fibres and the substance insoluble in water and alcohol, which appeared to be oxigenized extract, was composed of alumine and carbonate of lime.

This substance, being incinerated, left a whitish powder, soluble in great part with effervescence in muriatic acid. This solution contained alumine, lime, and a little iron. The portion not soluble in muriatic acid, being treated with potash, yeilded a siliceous precipitate on the addition of an acid.

The water employed to precipitate the resin left after evaporation poration a brown matter, weighing five parts, of a bitter taste, attracting the moisture of the atmosphere, soluble in alcohol, and copiously precipitated from its aqueous solution by acetate of lead. This substance exhibited all the properties of extract.

From this analytical essay therefore it follows, that Aleppo scammony is composed of

| | |
|---|---|
| Resin | 60 |
| Gum | 3 |
| Extract | 2 |
| Vegetable fibres, earthy matter, &c. | 35 |
| | 100 |

and that Smyrna scammony contains

| | |
|---|---|
| Resin | 29 |
| Gum | 8 |
| Extract | 5 |
| Vegetable fibres, &c. | 58 |
| | 100 |

Though the resins obtained from the two sorts of scammony have considerable analogy, yet, as that of Aleppo is yellow, transparent, and friable, while that of the Smyrna is darker coloured, and more difficult to powder, we thought it would not be useless to ascertain, whether there was any difference in their medicinal properties. In consequence several physicians undertook to make comparative trials on persons of nearly similar constitutions, but they have not yet observed any difference in their purgative effects.

From the preceding analysis we may conclude, that scammony is a true gum-resin mingled with a little extract. It is true it contains much less gum than the other gum-resins, yet enough to form a milky liquor with water.

The action of the alcoholic tincture of scammony on litmus led us naturally to examine, whether the property of reddening this blue colour was owing to an acid. None of our experiments having furnished a direct proof of this, we made a comparative trial of some resins, which we subjected to the following experiments.

1. *Sandarach*. The resin is converted into a grumous mass by boiling in water. The filtered liquor remains clear; and, when evaporated to a certain point, slightly reddens tincture of litmus; its taste is bitter; it does not alter infusion of violets; it is not precipitated by alcohol, or acetate of of lead, which shows, that it contains neither gum nor extract. It is therefore a pure resin.

The resin thus treated with boiling water was dissolved in alcohol. This tincture strongly reddened that of litmus, and had no action on sirup of violets.

Powdered sandarach was digested in alcohol, and boiling water poured into the hot filtered solution, which precipitated the resin. The filtered liquid grew turbid on cooling; it had the strong smell of resin of sandarach; its taste was bitter; and its action on the tincture of litmus was so weak, that it could not be supposed to contain a free acid.

2. *Mastic.* This substance exhibits nearly the same phenomena as the preceding; but the resin concretes into a mass in boiling, like turpentine. The water has a bitter taste, and has no action either on litmus or sirup of violets. The resin, on the contrary, strongly reddens tincture of litmus.

3. *Olibanum* forms with hot water a thick pap, which is separated from the liquid with difficulty even by filtration. This water has a blackish brown colour, is not precipitated by acetate of lead, and does not alter the colour of litmus; but alcohol throws down a copious precipitate from it, which proves, that this substance is composed of gum and resin.

The alcoholic tincture strongly reddens that of litmus.

If the resins that have most action on the colour of litmus be heated with all due precautions on a sand bath, no acid sublimes.

Treated with lime, according to Scheele's process, no calcareous benzoates are formed.

4. Lastly, the gum resin ammoniacum, myrrh, elemi, anime, galbanum, tacamahacca, resin of jalap, both prepared by ourselves and that of the shops, Venice turpentine, oil of turpentine, and several other resinous and gum-resinous substances, afforded the same results as those obtained from scammony, sandarach, and olibanum. From these facts it appears still difficult to solve the question, whether the reddening of litmus by resins be owing to the presence of an acid in them.

If acids alone had the property of reddening vegetable blues we should not hesitate to admit their existence in resins, though not yet otherwise demonstrated by experiment. As to the infusion of violets not being reddened by resins, this property occurs in the sublimed acid of benzoin, which strongly reddens infusion of litmus, but does not alter the colour of violets. Has this acid, notwithstanding its solubility in water, some analogy to resins? On this we refrain from giving a decided opinion; yet we are inclined to believe, that this substance is a compound of a vegetable acid and a small small portion of resin, to which perhaps its solidity is owing. Finally, as all the vegetable acids are soluble in water, it is difficult to ascribe to an acid this property in resins of reddening litmus. It appears more proper therefore, to consider the reddening of litmus as a character of resins, till fresh experiments have proved the contrary.

